# Retraction: Identification of novel candidate targets for suppressing ovarian cancer progression through IL-33/ST2 axis components using the system biology approach

**DOI:** 10.3389/fmolb.2025.1605599

**Published:** 2025-04-10

**Authors:** Frontiers Editorial Office

The Journal retracts the 2 June 2023 article cited above.

Following publication, concerns were raised regarding the contributions and affiliations of the authors of the article. Image duplication concerns were also identified with Figure 8. Our investigation, conducted in accordance with Frontiers policies, confirmed a serious breach of our authorship policies and of publication ethics.

This retraction was approved by the Chief Executive Editor of Frontiers. The authors received a communication regarding the retraction and had a chance to respond. This communication is recorded by the publisher.

